# Conceptualizing Contraceptive Agency: A Critical Step to Enable Human Rights-Based Family Planning Programs and Measurement

**DOI:** 10.9745/GHSP-D-23-00299

**Published:** 2024-02-28

**Authors:** Kelsey Holt, Sneha Challa, Phoebe Alitubeera, Lynn Atuyambe, Christine Dehlendorf, Christine Galavotti, Ivan Idiodi, Ayobambo Jegede, Elizabeth Omoluabi, Peter Waiswa, Ushma Upadhyay

**Affiliations:** a University of California, San Francisco, San Francisco, CA, USA.; b Makerere University School of Public Health, Kampala, Uganda.; c Bill & Melinda Gates Foundation, Seattle, WA, USA.; d Akena Plus Health, Abuja, Nigeria.

## Abstract

We propose a detailed framework for contraceptive agency to serve as a rights-based guide for centering individuals’ ability to make and act on their own contraceptive choices, regardless of what those choices are, in program design and evaluation.

## Key Messages

- Despite widespread agreement on the importance of a human rights-based approach to contraception programming, a gap exists between the principle of valuing individual choice and what programs are held accountable for in practice. This gap is reflected in the increasingly recognized need for new rights-based measures.
- We discuss 6 opportunities for conceptual and measurement innovation and propose a framework for the construct of “contraceptive agency”— the ability of an individual to make and act on decisions related to whether to do something to avoid or delay pregnancy and what, if anything, to do when they are not actively trying to become pregnant.
- The contraceptive agency framework can serve as a guide for centering individuals’ ability to make and act on their own contraceptive choices, regardless of what those choices are, in contraception program design and evaluation.

## BACKGROUND

The human right to control one’s own childbearing requires that individuals can both make and act on reproductive decisions.^1^ Thus, frameworks for ensuring a rights-based approach to contraception emphasize the importance of supporting individuals and couples to make and act on their own choices, regardless of what they choose.^2^^–^^4^ However, a gap exists between the importance of ensuring individuals can make their own contraceptive choices and act on them without interference in principle and what programs are held accountable for in practice, as reflected in a lack of robust rights-based measures and prioritization of contraceptive use focused, rather than rights-based, benchmarks of success.^5^^–^^9^

Several new frameworks and measures related to reproductive empowerment and autonomy have been published in the last decade and represent building momentum toward operationalizing a rights-based approach to supporting people’s reproductive decision-making.^10^^–^^13^ In this commentary, we review these frameworks and measures and argue that there remains a need for innovation in the conceptualization and measurement of the degree to which individuals have agency related to contraception. A focus on agency aligns with other recent global efforts calling attention to the often-overlooked importance of focusing on an individual’s capacity to make and carry out decisions related to their health and well-being—regardless of what they decide.^14^^–^^16^

In this commentary, we delineate specific conceptual gaps we seek to fill before presenting a detailed framework for the construct of contraceptive agency. Consistent with Donald et al.’s multidisciplinary definition of agency as the ability to define one’s goals and act on them^17^ and Sato et al.’s definition of agency as an individual’s ability to produce an effect in pursuit of goals and values,^18^ we define contraceptive agency as the ability of an individual to make and act on decisions related to whether to do something to avoid or delay pregnancy and what, if anything, to do when they are not actively trying to become pregnant. This definition draws attention to the degree of agency one has related to using or not using contraception to avoid or delay pregnancy and is agnostic to the decision itself (to use or not use contraception) in acknowledgment of the fact that there are legitimate reasons why people may make empowered decisions not to use contraception even when they also do not want to be pregnant.^9^ Note that agency related to other medical uses of contraception, such as management of acne or heavy periods, is beyond the scope of this definition.

After presenting the contraceptive agency framework, we discuss how the framework can be applied in the context of program design and evaluation, drawing on our experience with the Innovations for Choice and Autonomy (ICAN) project. ICAN’s focus is developing and testing approaches to implementing the self-injectable contraceptive technology in a way that meets women’s self-defined needs. As part of this work, we have used the contraceptive agency framework to guide development of self-injection programming and are developing and testing a new measure of contraceptive agency that we will use to evaluate the success of ICAN’s programming. Our team includes members from Nigeria, Uganda, and the United States, with diverse disciplinary backgrounds, expertise, and lived experiences that we have leveraged to collectively develop this contraceptive agency framework and our forthcoming contraceptive agency measure.

We present a contraceptive agency framework and discuss how the framework can be applied in the context of program design and evaluation.

## OVERVIEW OF FRAMEWORKS AND MEASURES RELATED TO CONTRACEPTIVE AGENCY

The Reproductive Autonomy Scale (14 items), published in 2014, was groundbreaking in providing a measure of “the power to decide about and control matters associated with contraceptive use, pregnancy, and childbearing.”^10^ Although this measure captures influence from in-laws or others, the authors focused primarily on relationships between women and their male partners, with items related to partner relationships in 3 domains: freedom from coercion, communication, and decision-making.

In 2018, the International Center for Research on Women (ICRW) Conceptual Framework for Reproductive Empowerment defined agency as the capacity of individuals to take deliberate actions to achieve their reproductive desires and preferences in 3 domains: voice, choice, and power.^11^ The authors note that the implication of this definition is that:

*individuals should be able to express their childbearing desires to their partners, providers, and others; meaningfully participate in communication and decision-making with partners, with providers, and within their communities; and shape desired outcomes related to marriage, the conditions of sexual intercourse and the use of contraception.*

The ICRW framework resulted in both the Reproductive Empowerment Scale (20 items)^19^ and the Reproductive Decision-Making Agency Measure (12 items), which captures the “degree to which individuals are meaningfully engaged in the decision-making process, and their level of satisfaction with their own influence over the decision itself.”^15^

Shortly after the ICRW framework was published, the Women’s and Girls’ Empowerment in Sexual and Reproductive Health (WGE-SRH) framework and WGE-SRH index (21 items) were published by the Performance Monitoring and Accountability project.^13^^,^^20^ The WGE-SRH framework outlines a 3-stage empowerment process comprising “existence of choice,” “exercise of choice,” and “achievement of choice,” referring to “contraceptive use by choice” as one of the specific manifestations of sexual and reproductive health (SRH) empowerment.^13^ The resulting WGE-SRH index includes several subscales that capture each stage.^20^

In 2020, Senderowicz published a framework for another highly related construct, contraceptive autonomy, defined as “factors necessary for a person to decide for themselves what they want in relation to contraception and then to realize that decision” with subdomains of informed choice (covering individuals’ knowledge of options), full choice (availability and affordability of method provision and removal services), and free choice (whether contraceptive use or nonuse is voluntary).^12^

In 2023, Harper and colleagues published a new measure of decision-making agency in the contraception clinical encounter; the measure was developed and validated in the U.S. context.^16^ The authors’ review of the empowerment and patient-centered care literature led them to define 3 domains to guide item generation for this new measure: freedom from coercion, nonjudgmental care, and active decision-making. The resulting scale is unidimensional and composed of 7 items that capture agency in the clinical encounter.

## OPPORTUNITIES FOR INNOVATION IN DEFINING AND MEASURING CONTRACEPTIVE AGENCY

These frameworks and measures have a common recognition of the importance of individuals’ ability to make and act on SRH decisions. We agree this duality is critical to include in any conceptualization of contraceptive agency and other constructs related to fulfillment of sexual and reproductive rights, and we seek to build on these foundational efforts in our conceptualization of a new contraceptive agency framework. In reviewing these related efforts and considering gaps in our ability to measure contraceptive agency, our team collectively identified 6 opportunities for innovation.

In our conceptualization of a new contraceptive agency framework, we seek to build on existing frameworks’ recognition of the importance of individuals’ ability to make and act on SRH decisions.

### Focus on Contraception-Specific Issues

The reproductive autonomy, ICRW, and WGE-SRH frameworks and tools are highly innovative in providing broader conceptualizations of empowerment—encompassing issues related to contraception, abortion, volitional sex, and pregnancy.^11^^,^^13^ This holistic vision is critical and provides a valuable approach for looking across sectors at how programs support SRH. At the same time, contraception-specific frameworks related to agency are lacking. Although Harper et al.’s agency scale is an important contribution to the field, it focuses specifically on contraceptive decision-making in the clinical encounter—only 1 component of agency related to contraceptive decisions and actions.^16^ Contraception-specific frameworks and resultant measures are arguably more actionable than broader reproductive empowerment-related frameworks as tools to promote rights-based family planning as they outline more specific contraception-related issues for intervention.

### Shift Away From Equating Covert Use With Disempowerment

The ability to engage others in decision-making has been equated with empowerment in the family planning field, as illustrated by the dominance of items on support from others or whether one can share opinions with partners and health care providers in the ICRW and WGE-SRH measures.^19^^,^^20^ This approach assumes all individuals desire others’ involvement in decision-making, which is not uniformly the case. Additionally, we argue that limited or no engagement from others does not inherently indicate lack of empowerment. The reproductive empowerment scale goes so far as to include items equating leveraging social support to convince partners to use contraception with empowerment,^19^ leaving out the possibility that an empowered person could choose to use contraception without telling anyone. In fact, while we recognize that disempowering restrictive social and gender norms are often to blame for covert use, we argue that covert use can in some cases reflect higher levels of agency. Covert use under these circumstances could indicate one’s assessment of the constraints in their environment and a deliberate decision to counter expectations and norms to act in accordance with their goals, as prior research has documented that some women do.^21^^,^^22^ Therefore, there is a need to incorporate this perspective in measures of contraceptive agency to allow for individual preferences for involving others. Notably, the reproductive decision-making agency measure is innovative in taking into consideration not just whether one shares their opinion with a partner but whether they want to share that opinion and how satisfied they are with the outcome.^15^

### Include the Extent to Which People Are Conscious of Their Rights and Societal Injustices

Agency in SRH decision-making tends to be narrowly conceptualized and measured with a focus on involvement and influence of others, as with the reproductive autonomy, reproductive empowerment, reproductive decision-making, and WGE-SRH frameworks,^10^^,^^19^^,^^20^ or a focus on knowledge and information, as with the contraceptive autonomy construct.^12^ In fact, agency in decision-making relies on a broader set of internal processes related to the formation of values-based preferences that have been understudied in the SRH field.^17^ Critical reflection is an important construct relevant to agency in decision-making. Borrowed from critical consciousness theory, critical reflection describes how people become aware of systemic social injustice as a precursor to taking action toward liberation.^23^ This suggests that it is important for contraception-related frameworks and measures to include the extent to which individuals recognize what their rights are and what societal injustices, such as gender inequities and poverty, may be constraining them. Though some previous efforts have engaged with the concept of critical consciousness in describing empowerment-related constructs, existing measures to capture those constructs lack items about critical consciousness as a part of decision-making—for example, critical consciousness is part of the reproductive empowerment framework but not the measure.^11^^,^^19^

### Broaden Acknowledgment and Measurement of Sources of Interference

Existing programs and measures primarily focus on partners as a key source of interference in women’s contraceptive agency. This is understandable, given inequitable gender norms and the documented prominence of partner influence on contraceptive use and decisions.^21^^,^^24^^–^^26^ However, there is a need to expand acknowledgment of other sources of social influence, particularly among adolescents for whom parental involvement is often more salient and people who live with extended family, including in-laws. Regarding measurement, the reproductive decision-making and WGE-SRH measures rely heavily on questions related to partner influence, making these measures not applicable for individuals without partners who may still face constraints to their contraceptive agency. The reproductive empowerment and reproductive autonomy measures include items assessing who makes the final decision about contraceptive use and include other family members and an “other” option in addition to partners, but these 2 measures rely heavily on partner-related items.^10^^,^^19^ In contrast, the agency in a contraceptive decision-making measure recently developed in the United States focuses exclusively on provider influence.^16^

There is a need to expand acknowledgment of other sources of social influence, particularly among adolescents for whom parental involvement is often more salient and people who live with extended family.

### Develop Universally Applicable Measures

Measures of empowerment and rights-fulfillment in the SRH field are predominantly developed for women, given their childbearing capabilities and the inequitable gender norms that have been shown to limit women’s rights and agency. At the same time, calls have been made for more attention to sexual and reproductive health and rights among men and gender nonbinary people.^27^^–^^30^ Inclusive concepts and measures that are not gender specific can support sexual and reproductive health and rights for all. This is particularly important as we experience a cultural shift toward recognizing shared responsibility for pregnancy prevention and family planning and the need for contraceptive methods that work for different bodies.

### Shift Away From Equating Contraceptive Use With Empowerment

Existing empowerment frameworks acknowledge that acting in accordance with one’s goals is the aim of contraceptive access efforts, yet measures resulting from these frameworks tend to rely on items that equate contraceptive use specifically with empowerment. For example, the reproductive empowerment measure subscale on social support includes 3 items about the extent to which individuals could convince an unsupportive partner to let them use contraception and no items about support for decisions not to use contraception.^19^ One notable exception is the contraceptive autonomy framework, which explicitly makes clear that items should assess whether contraceptive use is desired and removals for long-term methods are accessible.^12^

## CONTRACEPTIVE AGENCY FRAMEWORK

Considering the aforementioned opportunities for conceptual innovation, we drew on interdisciplinary theoretical constructs related to agency,^17^ health behavior,^31^ and critical consciousness^23^ to develop a framework delineating 8 specific constructs comprising contraceptive agency. Domain 1 covers constructs related to decision-making, and Domain 2 covers constructs related to acting on decisions (Box). These 2 theoretically informed domains cover the range of psychosocial constructs necessary for an individual who is not actively trying to get pregnant to have full agency over what (if anything) they decide to do related to avoiding or delaying pregnancy. A person of any gender with high levels of each of the 8 constructs is considered to have high contraceptive agency whether or not they decide to use contraception and regardless of what method they choose.

BOX Detailed Definition of Contraceptive Agency Domains

**Domain 1: Agency in Decision-Making Related to Avoiding or Delaying Pregnancy**

1. To **be clear about one’s personal values** related to doing or not doing something to avoid or delay pregnancy
2. To **have information and support in accordance with one’s preferences** to make choices about doing or not doing something to avoid or delay pregnancy
3. To **be conscious of the right to contraceptive choice, including:**
  - Entitlement to use, not use, switch, or stop using contraception
  - Right to have access to a wide range of contraceptive methods and to choose which method, if any, to use
4. To **exercise critical reflection** and be aware that intersecting social constructions can constrain or enhance individuals’ ability to exercise the right to choices related to doing or not doing something to avoid or delay pregnancy (critical reflection construct from critical consciousness theory^23^)
5. To **believe one has control over decisions** related to doing or not doing something to avoid or delay pregnancy, including who they would like to be involved in those decisions (perceived control construct from health behavior theory^31^)
6. To **have confidence in one’s ability to form and act on preferences** related to doing something or not doing something to avoid or delay pregnancy (self-efficacy construct from health behavior theory^31^)

**Domain 2: Agency in Acting on Decisions Related to Avoiding or Delaying Pregnancy**

7 To be able to **act in accordance with one’s preferences** related to doing or not doing something to avoid or delay pregnancy
8 To **have control over who and to what extent others are involved** in and aware of one’s preferred actions related to avoiding or delaying pregnancy

We recognize the inherent risk that defining individual-level constructs such as this one will primarily motivate behavioral rather than social or structural interventions. Thus, in the Figure, we situate contraceptive agency within the broader context of influences on individual decisions and actions, drawing on the Integrated Behavioral Model^31^ and the Social Ecological Model.^32^ On the left, we acknowledge the historical, political, economic, and cultural environment that influences and interacts with donors’, governments’, and nongovernmental organizations’ priorities related to family planning. Here, we also account for individual characteristics, such as religion, age, and ethnicity. Together, these individual, structural, and systemic factors form the context in which individuals make and act on decisions related to avoiding or delaying pregnancy. The contraceptive care environment (access to and quality of services including the accessibility, structure, and process of care^33^^,^^34^) and the social environment (including influence from community members, household and other family members, peers, partners, and the media^32^) are depicted on the top and bottom, respectively, as directly influencing contraceptive agency. In accordance with the Integrated Behavioral Model, we depict that the degree of how salient avoiding or delaying pregnancy is within the broader context of an individual’s life and priorities also can play a role in acting on one’s preferred approach (which may include using any form of contraception or not, even when one does not desire pregnancy).^31^

**FIGURE:**
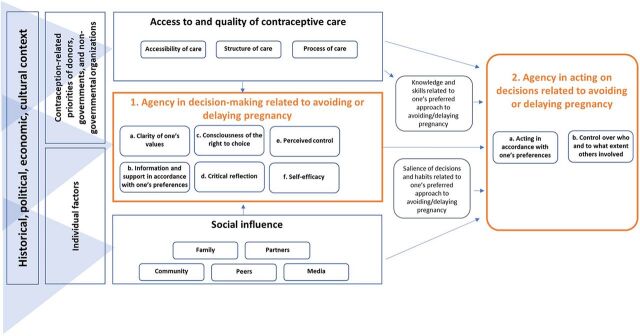
Contraceptive Agency Framework: Constructs Constituting and Influencing Agency

## IMPLICATIONS FOR PRACTICE AND MEASUREMENTS

The contraceptive agency framework can be applied as a practical resource to guide program design and evaluation. In the design phase, the 8 contraceptive agency constructs from the framework can serve as a menu of areas where programs can choose to intervene to improve agency. The framework will encourage programming that innovates beyond the common focus on knowledge and self-efficacy in many social and behavior change approaches^35^^,^^36^ toward a fuller set of activities that can promote contraceptive agency. For example, through the theoretically informed constructs of consciousness of the right to contraceptive choice and critical reflection about constraints to contraceptive choice, programming can be implemented to make sure individuals are aware of their rights and the conditions that constrain them in acting on those rights. The structural and social factors depicted in the framework as patterning uneven opportunities for agency based on social position will also guide program designers to recognize that agency is impacted by social influences at family, peer, partner, and community levels and that programs should strive to help individuals navigate these social and structural factors as much as is feasible without relying solely on individual-level solutions.

The framework will encourage programming that innovates beyond the common focus on knowledge and self-efficacy toward a fuller set of activities that can promote contraceptive agency.

Depending on objectives and resource constraints, programs will understandably prioritize specific constructs within the framework without needing to intervene with all elements. Indeed, the framework should serve as an aspirational guide and ensure donors and programs contend with the range of approaches necessary to comprehensively support and hold programs accountable for contraceptive agency. The framework can also be used as a tool to advocate for taking a rights-based approach by giving programs a concrete path toward prioritization of agency rather than other non-rights-based end goals, such as reducing fertility. We note that the framework is meant to complement rather than replace other tools to aid in program planning, such as those required for inventory planning or ensuring high-quality, rights-based service delivery.^37^

In the ICAN project, we used the contraceptive agency framework to guide the design of new programs to support diffusion of self-injection of subcutaneous depot medroxyprogesterone acetate (DMPA-SC). Recognizing the complexity and inherent tension in focusing on a single method while upholding informed choice, we used the contraceptive agency framework to guide our human-centered design process in Uganda toward solutions that would first and foremost support agency in making and acting on one’s own decisions rather than promoting uptake of self-injection. Our new community-based program entails leveraging peer social support to support women in making and acting on their own contraceptive decisions—whatever those decisions may be—while also diffusing information about the option of self-injection and having peers offer moral support for those interested in self-injecting (results forthcoming). Specific constructs from the contraceptive agency framework were used in the design of program materials. For example, peer support training materials discuss the importance of making sure women know their rights, are confident in their ability to make a choice, and have the level of support and information that they themselves desire. Self-injection is positioned as a method women should be aware of and supported to use if they desire, but the contraceptive agency framework helped us prioritize agency-related programming over an approach that prioritized promoting contraceptive uptake. Our forthcoming contraceptive agency measure (discussed next) will be used in the evaluation phase to gauge the success of these programs in upholding or promoting women’s agency, regardless of how many choose self-injection.

Related to measurement, the 8 constructs in the framework can serve as a guide for what programs should measure to ensure they promote agency related to contraceptive decision-making (Domain 1) and actions (Domain 2) and that they are doing no harm in those same areas. Our team is developing a new scale to measure contraceptive agency (results from research in Uganda, Nigeria, and the United States are forthcoming). The item pool for the new scale was developed based on formative research with women and the aforementioned gaps in other measures, such as a lack of items on the degree of support for decisions not to use contraception and the predominance of items that equate contraceptive nonuse or covert use with disempowerment. Recognizing that contraceptive agency is complex and that the resulting scale will likely be multidimensional, we envision that this new measure will be particularly well suited for application in program evaluations and other special studies. Subscales that comprise the measure can be applied alone or in tandem with other subscales, depending on program priorities, and we also plan to create a short form to facilitate the measure’s integration into regular program monitoring and, eventually, population-based surveys. Efforts to improve the measurement of complex constructs, such as quality of care and fulfillment of reproductive intentions, have required careful contextualization and direction.^38^^,^^39^ Thus, we recognize that publication of the new contraceptive agency scale will necessitate guidance on its application for a range of different contexts, including cross-sectional studies, program monitoring, and longitudinal studies.

## CONCLUSION

The contraceptive agency framework can complement existing rights-based program planning tools^37^ by helping programs prioritize intervention components that will support individuals’ ability to make and act on decisions related to avoiding or delaying pregnancy—regardless of what they choose. Our review of related frameworks confirms this is a novel contribution to the literature. Our call for a turn toward a focus on agency is in line with other recent global efforts that have emphasized the often-overlooked importance of agency.^14^^–^^16^ Programmatic and measurement innovations resulting from the contraceptive agency framework will be critical to help move the family planning field toward implementing—and holding ourselves accountable for—progress toward rights-based principles that are widely agreed upon in practice but less often operationalized and measured.^5^^–^^9^

## Notes

**Peer Reviewed**

**First published online:** February 13, 2024.
